# Antibodies to *Plasmodium vivax* reticulocyte binding protein 2b are associated with protection against *P*. *vivax* malaria in populations living in low malaria transmission regions of Brazil and Thailand

**DOI:** 10.1371/journal.pntd.0007596

**Published:** 2019-08-19

**Authors:** Wen-Qiang He, Stephan Karl, Michael T. White, Wang Nguitragool, Wuelton Monteiro, Andrea Kuehn, Jakub Gruszczyk, Camila T. França, Jetsumon Sattabongkot, Marcus V. G. Lacerda, Wai-Hong Tham, Ivo Mueller

**Affiliations:** 1 Infection and Immunity Division, Walter and Eliza Hall Institute of Medical Research, Parkville, Victoria Australia; 2 Department of Medical Biology, University of Melbourne, Melbourne, Australia; 3 Population Health and Immunity Division, Walter and Eliza Hall Institute of Medical Research, Parkville, Victoria, Australia; 4 Vector-borne Diseases Unit, Papua New Guinea Institute of Medical Research, Madang, Papua New Guinea; 5 Malaria Parasites and Hosts Unit, Department of Parasites & Insect Vectors, Institut Pasteur, Paris, France; 6 Department of Molecular Tropical Medicine and Genetics, Faculty of Tropical Medicine, Mahidol University, Bangkok Thailand; 7 Fundação de Medicina Tropical Dr. Heitor Vieira Dourado, Manaus, Amazonas, Brazil; 8 ISGlobal, Barcelona Centre for International Health Research (CRESIB), Hospital Clinic-Universitat de Barcelona, Barcelona, Spain; 9 Mahidol Vivax Research Unit, Faculty of Tropical Medicine, Mahidol University, Bangkok, Thailand; University of Sao Paulo, BRAZIL

## Abstract

**Background:**

The *Plasmodium vivax* Reticulocyte Binding Protein (PvRBP) family is involved in red blood cell recognition and members of this family are potential targets for antibodies that may block *P*. *vivax* invasion. To date, the acquisition of immunity against PvRBPs in low malaria transmission settings and in a broad age group of exposed individuals has not been investigated.

**Methodology/Principal findings:**

Total IgG antibody levels to six members of the PvRBP family (PvRBP1a, PvRBP1b, PvRBP2a, PvRBP2b, a non-binding fragment of PvRBP2c (PvRBP2cNB) and PvRBP2-P2) were measured in samples collected from individuals living in two regions of low *P*. *vivax* endemicity in Brazil and Thailand. In both settings, levels of total IgG to PvRBP1a, PvRBP2b, PvRBP2cNB, and PvRBP2P-2 increased significantly with age (rho = 0.17–0.49; P<0.001). IgG responses to PvRBP1a, PvRBP2b and PvRBP2cNB were significantly higher in infected individuals by using Wilcoxon’s signed-rank test (P<0.001). Of the six PvRBPs examined, only antibodies to PvRBP2b were associated with protection against clinical malaria in both settings.

**Conclusion/Significance:**

Our results indicate that PvRBP2b warrants further preclinical development as a blood-stage vaccine candidate against *P*. *vivax*. Total IgG responses to PvRBPs were also shown to be promising immunological markers of exposure to *P*. *vivax* infection.

## Author summary

*Plasmodium vivax* preferentially invades young red blood cells called reticulocytes. Successful parasite invasion relies on the interaction between parasite ligands with human red blood cell receptors. The *P*. *vivax* reticulocyte binding protein family (PvRBP) plays a role in the invasion process. The role of PvRBP in the natural acquisition of immunity to *P*. *vivax*, especially in low transmission settings, is poorly understood. Using samples from longitudinal cohort studies from regions of low *P*. *vivax* endemicity in Brazil and Thailand, we showed that antibody responses to PvRBP1a, PvRBP2b, and PvRBP2cNB increased with age and were boosted during infection. In addition, higher antibody levels to PvRBP2b were strongly associated with a lower risk of clinical episodes of *P*. *vivax*. Based on these observations, we propose that PvRBP2b warrants further preclinical development as a blood-stage vaccine candidate against *P*. *vivax*.

## Introduction

*Plasmodium vivax* is the most widespread human malaria parasite species [1]. Although 4% of estimated cases globally are caused by *P*. *vivax*, this proportion is 36% outside the African continent [2]. The majority of *P*. *vivax* infection occurs in the South-East Asia Region, which accounts for 58% of cases worldwide [2]. *P*. *vivax* accounts for more than 30% of total malaria cases in South-East Asia and 64% in the Americas. In some countries, such as Thailand and Brazil, *P*. *vivax* infection is responsible for 80% and 90% of malaria cases respectively [2]. As many countries progress towards the malaria elimination, *P*. *vivax* infection has emerged as one of the key challenges [3]. This is likely to be related to several biological and epidemiological characteristics of *P*. *vivax*. First, *P*. *vivax* infections typically exhibit low levels of blood-stage parasitemia, thus reducing the proportion of infections detected by light microscopy and rapid diagnostic tests. Furthermore, compared to *P*. *falciparum*, *P*. *vivax* develops in a wider temperature range [4]; and transmissible gametocytes emerge earlier in the blood circulation (frequently before the development of clinical symptoms), making this parasite highly transmissible [5]. Last but not least, *P*. *vivax* forms hypnozoites in the liver [6,7], which can reactivate weeks to months after the initial infection resulting in relapses. Hypnozoites are undetectable by any current diagnostic assay [5] but are the cause for the majority of relapsing *P*. *vivax* blood-stage infections [8]. In highly endemic regions, children acquire clinical immunity to *P*. *vivax* at a younger age compared to *P*. *falciparum*, leading to protection from clinical disease before adolescence [9]. In low-transmission settings reduced exposure leads to all age groups being at risk of clinical *P*. *vivax* illness [9].

Invasion of red blood cells by *Plasmodium* merozoites depends on specific interactions between parasite ligands and red blood cell receptors [10]. *P*. *vivax* preferentially invades young red blood cells called reticulocytes. In particular, *P*. *vivax* prefers to invade reticulocytes with high levels of transferrin receptor 1 (TfR1 or CD71) [11]. To date, there are two known families of parasite adhesins that bind red blood cells: the *P*. *vivax* Duffy binding protein family (PvDBP) and the *P*. *vivax* reticulocyte binding protein family (PvRBP). The PvDBP family has at least three members: PvDBP, PvDBP2, and *P*. *vivax* erythrocyte binding protein (PvEBP) [12,13]. The PvRBP family has at least 11 members, encoded in three partial genes and eight full-length genes [12,14]. Analyses of the red blood cell binding specificity of native and recombinant fragments of PvRBP proteins show that different members of this protein family exhibit different binding preferences for normocytes and/or reticulocytes [15–19]. The crystal structures of the N-terminal domains of PvRBP2a and PvRBP2b have been recently determined, revealing a similar structural scaffold to that of *P*. *falciparum* reticulocyte-binding protein homolog 5 (PfRh5) [20–22], one of the leading vaccine candidates for *P*. *falciparum* blood stages [23].

The first receptor identified for *P*. *vivax* invasion was the Duffy Antigen Receptor for Chemokines (DARC) which binds to *P*. *vivax* PvDBP [24]. Historically, PvDBP-DARC binding was thought to be the main interaction for *P*. *vivax* invasion [25], but the identification of *P*. *vivax* infection in Duffy-negative patients suggest the presence of alternative invasion pathways [26]. Recent studies show that PvRBP2b binds to transferrin receptor 1 (TfR1) to mediate a critical invasion pathway for entry into reticulocytes [21,27]. Mouse monoclonal antibodies raised against PvRBP2b abolished the PvRBP2b binding to reticulocytes and inhibit *P*. *vivax* entry into reticulocytes [21]. These observations highlight the value of PvRBP2b as a potential vaccine candidate for *P*. *vivax*.

Whole genome sequence of *P*. *vivax Salvador* I has identified 10 *PvRBP* genes, and they are named as *PvRBP1a*, *PvRBP1b*, *PvRBP1* (Partial), *PvRBP2a*, *PvRBP2b*, *PvRBP2c*, *PvRBP2-P1*, *PvRBP2-P2*, *PvRBP2d*, and *PvRBP3* [14]. Genetic diversity of *PvRBP1a* is markedly lower than that of *PvRBP2c* from parasite isolates originating from Brazil and Thailand [28]. High diversity of *PvRBP2a* was observed among 31 *P*. *vivax* isolates, which includes 22 isolates from PNG, four from Thailand, and five reference strains [20]. The highest nucleotide diversity is concentrated in the first 2.2 kb with evidence of significant balancing selection in this region [20]. Genetic variation of *PvRBP2b* has been analyzed using 148 *P*. *vivax* samples in the MalariaGEN *P*. *vivax* Genome Variation project [21,29]. Balancing selection was observed within the N-terminal domain at amino acid positions 169 to 470 [21]. Signatures of balancing selection may be linked to genes encoding surface exposed-proteins of the invasive blood-stage merozoite [30], suggesting the possibility that PvRBPs are targets of immunity.

Several studies have examined different aspects of naturally acquired immunity to PvRBPs [16,18,31–33]. Immunoreactivity to five recombinant fragments of PvRBP1a was determined among 297 donors from three communities of Rondônia, a state in the western Amazon region of Brazil where *P*. *vivax* accounts for 70% of all malaria cases [31]. These results show that IgG responses towards these recombinant PvRBP1a fragments were age-independent but exposure-dependent. The positive correlation between IgG to PvRBP1a and previous malaria exposure was further supported by another study on 253 individuals from the same region [32]. In a separate study in Thailand, IgG levels to five different PvRBPs and their associations with age and parasitemia were investigated in 41 plasma samples from *P*. *vivax* infected adult patients [33]. Antibodies to all five PvRBPs increased with age and a negative correlation with *P*. *vivax* parasitemia was observed for antibody responses to PvRBP2b [33]. A study performed with 104 residents of the Republic of Korea showed no significant associations were found between humoral immune responses to PvRBP1a and PvRBP1b and parasitemia [18]. Collectively, these cross-sectional studies included only limited clinical information thus the association of immune responses to PvRBPs with risk of clinical *P*. *vivax* malaria can not be determined.

A recent study in a well-characterized longitudinal cohort of young children (1–3 years old) from an area of high malaria endemicity in Papua New Guinea (PNG) showed that antibodies to PvRBP1a and PvRBP2b were strongly associated with reduced risk of clinical malaria episodes as defined by fever plus parasitemia >500/μL [16]. A full understanding of the development of immune responses to PvRBPs in older populations and those living in areas of low endemic settings is still lacking. To better understand the development of immunity to PvRBPs, it is important to determine if protective immunity develops in regions of low *P*. *vivax* transmission or whether infections occur too infrequently to allow the development of protective immunity. In this study, we investigated the association of antibody responses to six PvRBPs with age, infection status, malaria exposure and clinical episodes in samples collected in Brazil and Thailand in 2013. Our results show that naturally acquired immunity to PvRBP2b could serve as a valuable correlate of protective immunity against clinical *P*. *vivax* episodes in all age groups from low transmission intensity settings.

## Methods

### Ethical statement

Ethics approvals were obtained from the Ethics Committee of the Faculty of Tropical Medicine, Mahidol University (MUTM 2013-027-01) and the Fundação de Medicina Tropical Doutor Heitor Vieira Dourado (349.211/2013).

### Cohort studies

Plasma samples used in the current study were collected as part of two longitudinal cohort studies conducted in Brazil and Thailand. The study in Brazil was undertaken in the villages of Brasileirinho, Puraquequara, and Ipiranga located in peri-urban areas of Manaus in April 2013 to May 2014 and included a total of 1274 participants of age ranges from 4 days to 102 years (Monteiro, Karl et al., in preparation). The study in Thailand was undertaken in the provinces of Kanchanaburi and Ratchaburi in May 2013 to June 2014 and included 1000 individuals aged from 1 to 82 years [34]. The two studies had a similar longitudinal design. In brief, participants were followed for 12 months and screened at monthly intervals for symptomatic illness and infection status as detected by PCR. All *P*. *vivax* infections were genotyped using PCR, allowing for the calculation of the incidence of genetically distinct blood-stage infections during follow-up (i.e., the molecular force of blood-stage infections, _mol_FOB) as previously described [35]. Plasma samples collected at the start of these studies were used for antibody measurement.

### Expression and purification of recombinant PvRBPs

The six PvRBPs used in this study were PvRBP1a (amino acid [aa] 160–1170), PvRBP1b (aa 140–1275), PvRBP2a (aa 160–1135), PvRBP2b (aa 161–1454), PvRBP2c Non-Binding (PvRBP2cNB, aa 501–1300) and PvRBP2P-2 (aa 161–641). Their accession numbers are PVX_098585, PVX_098582.1, PVX_121920, PVX_094255, PVX_090325, and PVX_101590, respectively (www.plasmodb.org/plasmo). Methods for protein expression and purification of have been described elsewhere [20,21].

### PvRBP conjugation and measurement of IgG responses

Conjugation of recombinant PvRBPs to Luminex 5.6 polystyrene microspheres was performed as previously described [36]. The corresponding concentrations of each of the six PvRBP constructs used to conjugate 2.5 x 10^6^ microspheres were as follows: PvRBP1a = 3.0 μg/mL; PvRBP1b = 11.4 μg/mL; PvRBP2a = 6.7 μg/mL; PvRBP2b = 0.2 μg/mL; PvRBP2cNB = 0.8 μg/mL; PvRBP2-P2 = 5.4μg/mL.

The multiplex antibody detection assay was performed as described elsewhere [16,36]. Briefly, plasma samples were diluted into 1:100 in phosphate-buffered saline containing 1% bovine serum albumin and 0.05% Tween. Samples were incubated with the conjugated beads (1:50 ratio) for 30 minutes under constant agitation. PE-conjugated donkey anti-human IgG fragment crystallisable region (Fc) was used as the secondary antibody in a volume of 100 μL/well at 1 μg/mL. Beads were read on a Bio-Plex200 reader. Results are reported as median fluorescence intensity (MFI). A blank well without plasma was included for determination of the true fluorescence background. As immune responses to pooled serum (from highly malaria exposed Papuan New Guinean adults (>18 years) from the Madang (n = 10) and East Sepik Provinces (n = 10)) are very strong, in each batch, a twofold serial dilution from 1/50 to 1/25,600 of this pool was included to generate a standard curve to minimize the variation from different plates.

### Statistical analyses

Specific standard curves from each Luminex assay plate were used for transformation of MFIs into relative antibody units (expressed as dilution factors that range from 1.95 x 10^−5^ or 1/51200 to 0.02 or 1/50) using a five parametric logistic regression model as described previously [16].

Statistical analyses were performed using STATA version 12 (StataCorp) and R version 3.2.1 (htpp://cran.r-project.org). LOESS smoothing with 95% prediction interval was applied to plot antibody levels in relation to age. Spearman’s rank correlation was used to assess associations between antibody levels and age and correlations among antibody responses against different antigens. The differences in antibody levels between categorical variables were assessed using Unpaired Two-Sample Wilcoxon’s Signed-Rank Test. Differences in the mean and proportions of population demography were evaluated by t-test and chi-square test. Multivariable logistic regression was used to investigate the association of current infection and infection in the follow-up periods with antibody responses, including the following variable as covariates for both studies: age, _mol_FOB, gender and pregnancy, where gender and pregnancy were combined into a categorical variable as below: non-pregnant female (reference group), pregnant female and male. As the Thai cohort was conducted on the border of Thailand and Myanmar—where cases are known to often be introduced from Myanmar, we also included the proportion of indoor-spraying and sleeping in Myanmar as covariates for the Thai cohort analyses. Receiver operating characteristics (ROC) curves were applied to determine the relative accuracy of antibody responses to each PvRBPs to predict current infection. Overall accuracy of each antigen to predict current infection was quantified as area under the curves (AUCs). AUCs were calculated on a threshold basis: if an individual’s baseline antibody levels were higher than a particular threshold, then that individual was classified as having concurrent infection. Cox proportional hazards models were used to investigate associations between antibody responses and time to first clinical episode. For this, a clinical episode was defined as the presence of axillary temperature ≥37.5°C or fever in the last 48 hours in the presence of any *P*. *vivax* density by light microscopy [37]. Antibody levels were divided into higher and lower groups based on the median values for the corresponding antigens and Kaplan-Meier estimates were computed until endpoints of the longitudinal cohort study were reached. Log-rank tests were used to explore the differences between survival curves.

All datasets were available in the Dryad repository: http://doi:10.5061/dryad.p678q83 [38].

## Results

### Demography of the study population and acquisition of antibodies to PvRBPs in Brazilian and Thai cohort studies

The prevalence of *P*. *vivax* infection by PCR at baseline for the Brazilian and Thai cohorts was 3.4% (39/1163, 95% CI 2.5%-4.6%) and 2.9% (28/973, 95% CI 2.0%-4.1%) respectively. Baseline characteristics of the study participants with infection are shown in S1 Table. In the Brazilian study, older individuals had higher risk of *P*. *vivax* infection (P = 0.035). Pregnant women were also more likely to have *P*. *vivax* infection (P = 0.004). In comparison with the general female population, males were more likely to be infected with *P*. *vivax* although only borderline significance was observed (P = 0.085). The participants with higher molFOB values were more likely to have infection (P<0.001). For the Thai study, participants with higher molFOB values, and males were more likely to be infected with *P*. *vivax* (P≤0.006). The age distribution and proportion of the population using insecticide-treated bed nets were similar for the two groups in both two studies (P = 0.148–0.789). The individuals who had travelled and stayed over night in in Myanmar was significantly higher in the infected population than in the non-infected population (P<0.001). On the other had, participants benefited from indoor spraying as a higher proportion of population in the uninfected group use this control strategy (P = 0.049).

**Fig 1:**
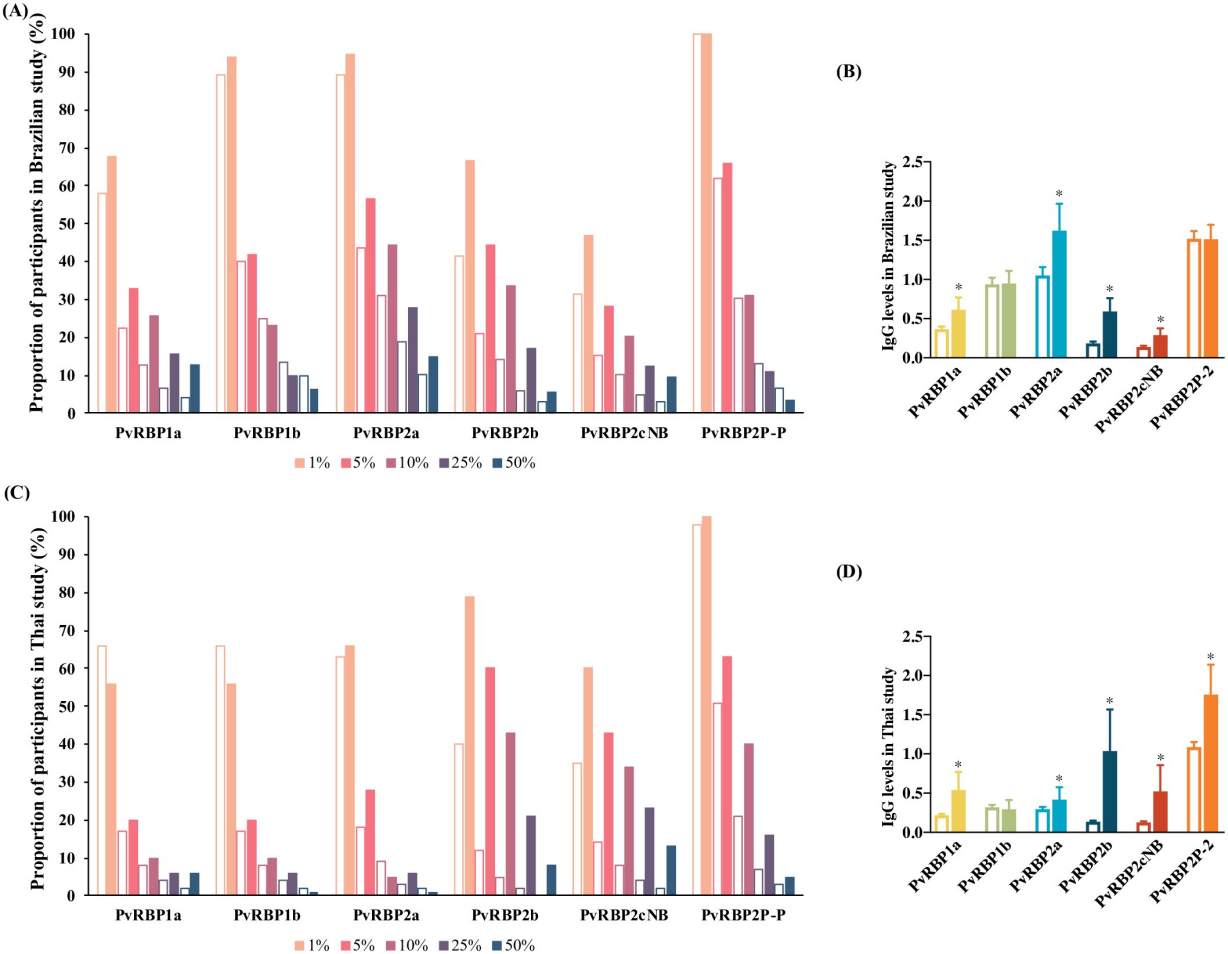
Antibody levels of Brazilian and Thai cohort studies in comparison with antibody levels of PNG adults. The comparisons of proportions of participants with IgG levels equivalent to >1%, >5%, >10%, >25%, and >50% of PNG adults levels between the uninfected individuals and those suffered any *P*. *vivax* infection for the longitudinal study periods were shown in A (Brazilian study) and C (Thai study). The empty bars represented the proportions of uninfected individuals and the filled bars displayed the proportions of individuals with any infection for each level (>1%, >5%, >10%, >25%, and >50%). Figure B and D showed the comparison of antibody levels between the uninfected and infected population for each antigen in the two cohort studies. Data were geometric mean with 95% confidence interval. IgG levels multiplied by 1000. The blank bars were the population without infection for the studies (n = 235 and n = 80 in Brazilian and Thai cohorts, respectively) and filled bars were those with any infection (n = 928 and n = 893 in Brazilian and Thai cohorts, respectively). *P<0.001 for the comparison of the two groups for antibody levels to each PvRBPs.

In this study, we used pooled plasma from immune PNG adults as a positive control and determined the number of study participants with IgG levels equivalent to >50%, >25%, >10%, >5%, or >1% of PNG adults’ IgG levels for both uninfected individuals and those with *P*. *vivax* infection during the period of the longitudinal study (Fig 1A and 1C, S2 Table). For antibody responses to PvRBP1a, PvRBP2b, and PvRBP2cNB in both studies, the prevalence of individuals with >1%, >5%, 10%, >25% and >50% of immune PNG adult levels among individuals with any infection were also higher than those without any infection (Fig 1A and 1C, S2 Table). The geometric mean antibody levels of participants with any *P*. *vivax* infection were higher than participants without any infection for the three antigens (P<0.001) (Fig 1B and 1D, S2 Table). Overall, apart from antibody levels of PvRBP2cNB (P = 0.137), Brazilian participants had higher antibody levels than Thai participants (P≤0.002), with the exception of antibody levels to PvRBP2cNB (P = 0.137).

### Total IgG in relation to age, infection and _mol_FOB

**Fig 2:**
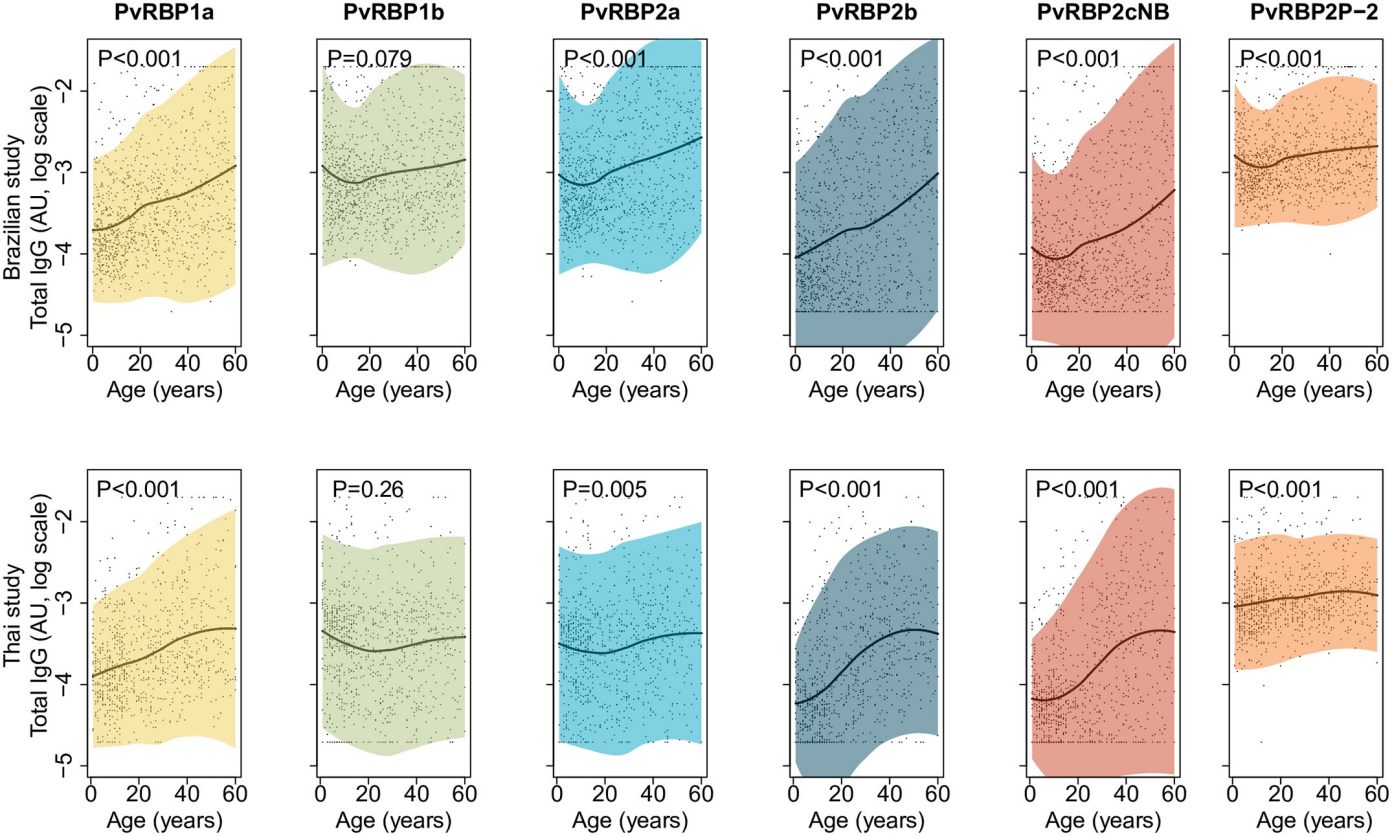
Antibody levels of IgG to six PvRBPs in relation to age. The top and bottom panels represent total IgG levels to six PvRBPs in relation to age of Brazilian and Thai participants, respectively. The line shows the LOESS smoothed estimate of the log_10_ transformed arbitrary unit (AU) and shaded regions represent 95% prediction interval. The black dots in the background represented the raw data. The Y-axis represents antibody levels in log_10_ transformed arbitrary units and the x-axis represents the age of participants.

The correlations of antibody responses to the six PvRBPs and age were determined. Levels of antibodies to PvRBP1a, PvRBP2b, PvRBP2cNB, and PvRBP2P-2 increased with age in both Brazilian and Thai studies (rho = 0.09–0.49; P≤0.005, Fig 2).

**Fig 3:**
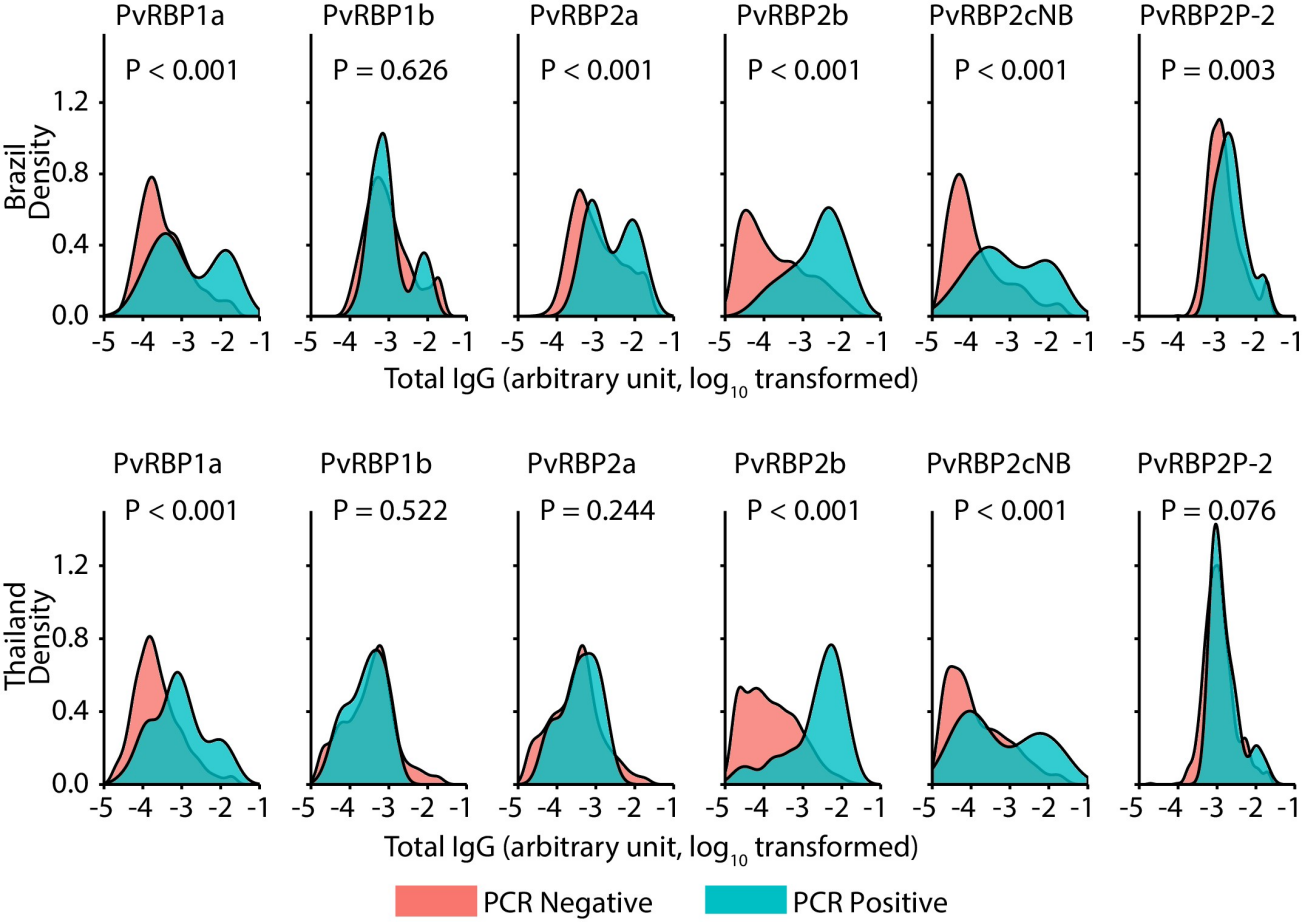
Distribution of antibody levels of PvRBPs and comparison of antibody levels between PCR positive and negative *P*. *vivax* population. Probability density functions were plotted in the figure divided by infection status, in which red refers to individuals without infection and blue refers to individuals with infection population (39 in Brazilian and 28 in Thai study). Y-axis represents density of abundance of antibody responses, x-axis represents levels of antibody responses; sample size (1163 in Brazilian and 973 in Thai study); P value for assessing significant differences between antibody levels in the PCR negative and PCR positive groups were calculated using Unpaired Two-Sample Wilcoxon’s Signed-Rank Test. P<0.001 in both cohorts for PvRBP1a, PvRBP2b and PvRBP2cNB.

Individuals with concurrent *P*. *vivax* infection by PCR at baseline (n = 39 in the Brazilian study; n = 28 in the Thai study) had significantly higher antibody levels to PvRBP1a, PvRBP2b, and PvRBP2cNB (P<0.001) (Fig 3).

**Fig 4:**
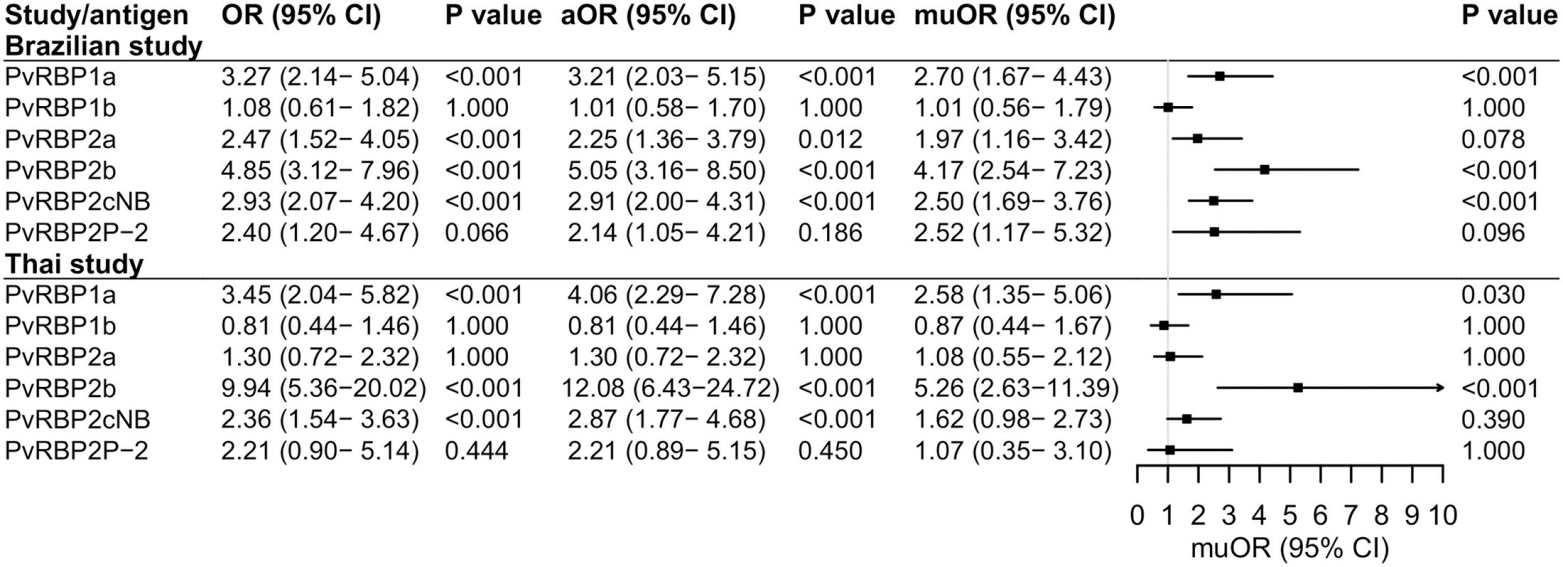
Associations of antibody responses to the six PvRBPs with concurrent infection of *P*. *vivax*. A logistic regression model was used to estimate the odds of a concurrent infection with every log rise in antibody levels. For the age adjusted model, only age was included as a covariate while for the age and exposure adjusted model (shown as muOR), the variables were included as below: age, sex, pregnancy, molFOB for both studies, and also the proportion sleeping in Myanmar and proportion of indoor spray in Thai cohort. The squares represent odds ratios (ORs) and the horizontal lines depict corresponding 95% confidence interval (CI). ORs>1 indicate that antibody responses are associated with increased risk of infection whereas ORs<1 indicate lower risk of infection. The ORs equal to 1 for the vertical line, which means there is no difference for the compared two groups. Abbreviations: OR, odds ratio; CI, confidence interval; aOR, age adjusted odds ratio; muOR, multivariable adjusted odds ratio.

Antibodies to PvRBP2b showed the strongest association with likelihood of infection in both studies for our unadjusted and adjusted analysis (Fig 4). For each unit increase in antibody levels (one log10-transformed antibody unit), it was associated with about 4-fold (muOR = 4.17, IC_95_ [2.54–7.23], P < 0.001) and 5-fold (muOR = 5.26, IC_95_ [2.63–11.39], P < 0.001) increase in the odds of being infected in the Brazilian and Thai studies, respectively. The accuracy of these predictions was further confirmed by receiver operating characteristic analyses, with AUC PvRBP2b being the highest for the prediction of concurrent infection (AUC = 0.824–0.865), followed by PvRBP1a (AUC = 0.713–0.733) and PvRBP2cNB (AUC = 0.695–0.784) (S1 Fig).

**Fig 5:**
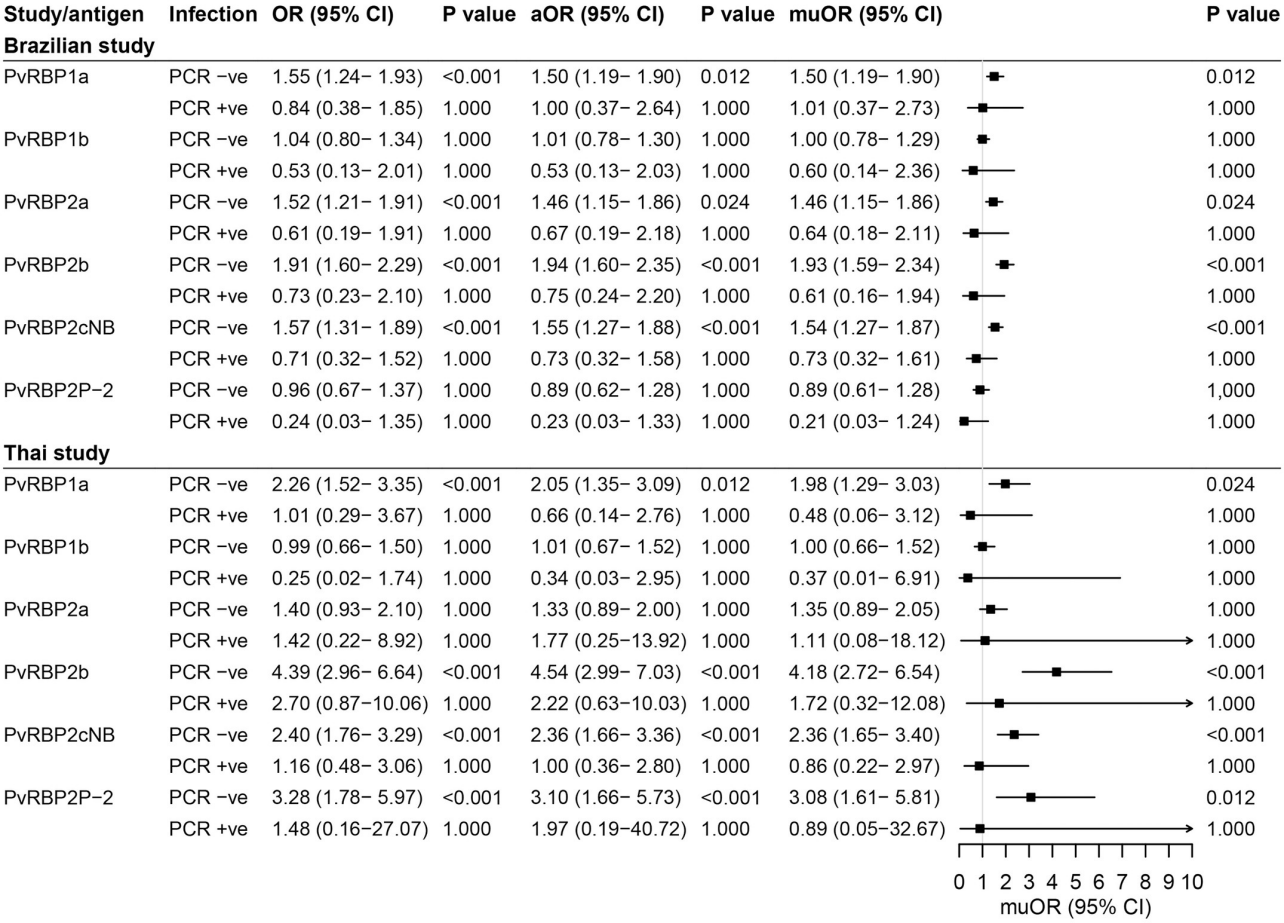
Associations of antibody responses to the six PvRBPs with following infections of *P*. *vivax* over the follow-up periods. A logistic regression model was used to estimate the odds of a following infection with every log rise in antibody levels. For the age adjusted model, only age was included as a covariate while for the age and exposure adjusted model, the variables were included as below: age, sex, pregnancy, molFOB, for both studies, and also the proportion sleeping in Myanmar and proportion of indoor spray in the Thai cohort. The above model was stratified by the baseline infection status. The squares represent coefficient and the horizontal lines depict corresponding 95% confidence interval. ORs>1 indicates that antibody responses are associated with increased risk of infection whereas ORs <1 indicates lower risk of infection. ORs equals to 1 for a vertical line. Abbreviations: OR, odds ratio; CI, confidence interval; aOR, age adjusted odds ratio; muOR, multivariable adjusted odds ratio.

To investigate the association of antibody responses to six PvRBP and risk of infection in the follow-up periods, we evaluated the association between antibody levels at enrolment and the risk of *P*. *vivax* infections over the 12 months of follow-up. In both studies, in the participants free of infection at enrolment, antibody responses to PvRBP1a, PvRBP2b, and PvRBP2cNB (and those to PvRBP2a in the Brazilian cohort only) were strongly associated with increased risk of infection over the study preriod for both unadjusted and asjusted models (P≤0.002, Fig 5). No difference was observed for individuals with infection at enrolment (Fig 5).

### Antibody responses to PvRBP2b predict reduced incidence of symptomatic malaria

**Fig 6:**
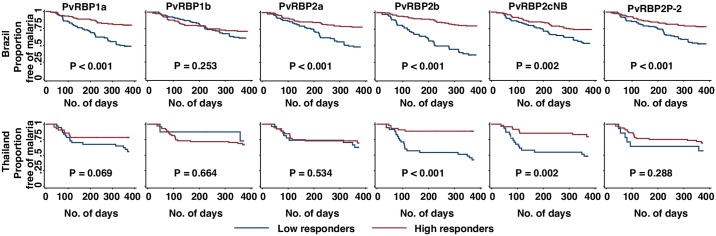
The association of antibody responses to PvRBPs and time to first *P*. *vivax* clinical episode. Kaplan-Meier curves showed time to first *P*. *vivax* clinical episode for total IgG responses against six PvRBPs in Brazilian and Thai studies. Antibody responses were equally divided into two groups: high (red line) and low (blue line) antibody reactivity, with a log-rank test to analyze the differences between every two groups. Only individuals experienced at least one asymptomatic infection during follow-up were involved in this analysis. Symptomatic *P*. *vivax* episode was defined as the presence of axillary temperature ≥37.5°C or fever in the last 48 hours in the presence of any *P*. *vivax* density by light microscopy.

For the Brazilian and Thai cohorts, a low burden of *P*. *vivax* disease was observed during follow-up, with only 6.4% (75/1163) and 2.6% (25/973) of participants experiencing at least one symptomatic episode of *P*. *vivax* malaria, respectively. In order to determine the association of antibody levels with protection against clinical episodes, we restricted our analyses to individuals with at least one infection during follow-up (Table 1), of which 31.8% (75/236) of the Brazilian and 31.6% (25/79) of the Thai cohorts experienced at least one symptomatic episode. Individuals were divided into high and low responder groups by median levels of IgG corresponding to each antigen. By using Kaplan Meier curves and log-rank test, in the Brazilian cohort, high responders to all PvRBPs except for PvRBP1b showed significantly longer time to develop symptomatic disease as compared to low responders (P≤ 0.002, Fig 6). In the Thai study, participants with higher antibody levels to PvRBP2b and PvRBP2cNB were also more likely to remain free of clinical episodes for longer periods than low responders (P≤0.002, Fig 6).

**Table 1:** Association between antibodies responses to PvRBPs and risk of clinical malaria.

Study	Antigen	Age-adjusted analysis		Exposed-adjusted analysis		Multivariate analysis	
		aHR* (95% CI)	P value	age+expHR** (95% CI)	P value	muHR*** (95% CI)	P value
Brazilian study	PvRBP1a	0.47 (0.31, 0.7)	**<0.001**	0.47 (0.31, 0.70)	**<0.001**		
	PvRBP1b	0.58 (0.36, 0.94)	0.156	0.59 (0.37, 0.95)	0.180		
	PvRBP2a	0.45 (0.31, 0.67)	**<0.001**	0.45 (0.30, 0.67)	**<0.001**		
	PvRBP2b	0.44 (0.33, 0.59)	**<0.001**	0.43 (0.32, 0.58)	**<0.001**	0.43 (0.32, 0.58)	**<0.001**
	PvRBP2cNB	0.51 (0.36, 0.71)	**<0.001**	0.50 (0.36, 0.70)	**<0.001**		
	PvRBP2P-2	0.27 (0.14, 0.53)	**<0.001**	0.25 (0.13, 0.51)	**<0.001**		
Thai study	PvRBP1a	0.51 (0.25, 1.00)	0.312	0.73 (0.35, 1.49)	1.000		
	PvRBP1b	1.42 (0.79, 2.55)	1.000	1.41 (0.70, 2.83)	1.000		
	PvRBP2a	0.95 (0.49, 1.83)	1.000	1.13 (0.52, 2.43)	1.000		
	PvRBP2b	0.34 (0.21, 0.55)	**<0.001**	0.50 (0.26, 0.94)	0.186	0.50 (0.26, 0.94)	**0.031**
	PvRBP2cNB	0.50 (0.30, 0.81)	**0.030**	0.65 (0.34, 1.24)	1.000		
	PvRBP2P-2	0.51 (0.16, 1.59)	1.000	0.65 (0.21, 2.02)	1.000		

Note. Antibodies levels were categorized into high and low responders by median levels to each detected antigen. Hazards ratio (HR) was obtained by using Cox proportional hazards model to compute the hazards between higher and lower groups. Only 236 and 79 participants from Brazil and Thailand studies were involved in the above analysis, of which 31.8% (75/236) and 31.6% (25/79) experienced a first symptomatic episode. Abbreviation: HR = hazard ratio; aHR = adjusted hazard ratio; muHR = multivariable adjusted hazard ratio.

*Adjustment for the age of participants.

**Adjustment for individual differences in exposure (molFOB), age, gender, and pregnancy for both studies, as well as the proportion of sleep in Myanmar and proportion of indoor spray in the Thai study. P values was corrected by multiplying a factor of 6.

***Multivariable analysis of antibodies univariately associated with protection by using stepwise backward selection with P value 0.05 as cutoff. Symptomatic *P*. *vivax* episode was defined as the presence of axillary temperature ≥37.5°C or fever in the last 48 hours in the presence of any *P*. *vivax* density by light microscopy.

Using Cox proportional hazards model, two separate models were tested by including age and other potential confounders (Table 1). Antibody responses to all the six antigens were significantly associated with reduced risk of clinical *P*. *vivax* malaria in the Brazilian study (_age+exp_HR = 0.25–0.59, P≤0.031, Table 1). In contrast, a protective association was only identified for PvRBP2b in the model adjusting age and other exposure variables in the Thai study (_age+exp_HR = 0.50, IC_95_ [0.27–0.94], P = 0.031). In addition, our multivariable model including antibodies univariately associated with protection revealed that revealed only antibody levels to PvRBP2b were associated with protection for both cohorts by using stepwise backwards selection (for Brazilian study: _mu_HR = 0.43, IC_95_ [0.32–0.58], P<0.001; for Thai study: _mu_HR = 0.50, IC_95_ [0.26–0.94], P = 0.032, Table 1). These results suggested that the significant association in the univariate model might be due to the strong correlation between PvRBP2b with other PvRBPs in Brazilian study (rho = 0.47–0.77, Table 2) and the correlation between antibody responses to PvRBP2b and PvRBP2cNB (rho = 0.76) in Thai study.

**Table 2:** Correlation between antibody responses to PvRBPs in the two cohort studies.

Study	Antigen	Correlation coefficient					
		PvRBP1a	PvRBP1b	PvRBP2a	PvRBP2b	PvRBP2cNB	PvRBP2P-2
Brazil study	PvRBP1a	1.00					
	PvRBP1b	0.37	1.00				
	PvRBP2a	0.62	0.77	1.00			
	PvRBP2b	0.65	0.47	0.77	1.00		
	PvRBP2cNB	0.64	0.52	0.79	0.77	1.00	
	PvRBP2P-2	0.42	0.73	0.66	0.50	0.54	1.00
Thailand study	PvRBP1a	1.00					
	PvRBP1b	0.33	1.00				
	PvRBP2a	0.51	0.88	1.00			
	PvRBP2b	0.59	0.35	0.52	1.00		
	PvRBP2cNB	0.64	0.39	0.58	0.76	1.00	
	PvRBP2P-2	0.40	0.52	0.56	0.43	0.45	1.00

Note. Correlation coefficient was determined by Spearman’s method. All P values was corrected by multiplying a factor of 6. All correlations are significant at a corrected P value of <0.001.

## Discussion

In this study, we characterized the association between IgG responses to six PvRBPs with *P*. *vivax* infection and clinical episodes in regions of low *P*. *vivax* endemicity in Brazil and Thailand. Our results suggest that IgG responses to PvRBP1a, PvRBP2b, and PvRBP2cNB could be immunological markers of asymptomatic *P*. *vivax* infection in these malaria low transmission areas. Furthermore, antibody levels to PvRBP2b showed strong association with protective immunity against clinical *P*. *vivax* episodes.

It has been reported that acquired clinical immunity to *P*. *vivax* malaria depends on repeated exposure to infection [39]. Asymptomatic infections seem to have boosted the total IgG antibody levels to PvRBP1a, PvRBP2b, and PvRBP2cNB. Both Brazilian and Thai study sites showed that antibody levels to these antigens increased throughout childhood and into adulthood, supporting the premise that antibodies to PvRBPs are a strong reflection of both concurrent and past exposures to *P*. *vivax* infections. In both Brazil and Thailand, we did not observe any association of antibody levels against PvRBP1b with age or cumulative exposure. However, antibody responses to PvRBP1b were associated with age and cumulative exposure in the young PNG children [16]. This is likely a result of the high transmission intensity in the PNG paediatric cohort, in which each child on average had acquired 14 new *P*. *vivax* blood-stage infections per year [35]. In our study, the prevalence of infection was only about 3%, which was reflected in the lower antibody levels to PvRBPs observed in both the Brazilian and Thai population in comparison to PNG children [16].

_mol_FOB estimates the number of distinct *P*. *vivax* clones acquired over the course of the follow-up period and is thus a direct measure of the individual heterogeneity in exposure to *P*.*vivax* [35]. A previous study indicates that _mol_FOB is an important predictor of clinical disease and a surrogate marker of malaria transmission [40]. Consistent with previous findings from PNG children living in a highly endemic *P*. *vivax* transmission region [16], we identified strong associations of antibody responses to PvRBP1a, PvRBP2b, PvRBP2cNB and infection in the follow-up periods in participants without concurrent infection. The boost in antibody levels by an active infection may have obscured similar associations in infected individuals. Nevertheless, our results suggest that antibody responses to PvRBP1a, PvRBP2b, and PvRBP2cNB may not only be markers of recent past infections but could potentially be used to predict the individuals with the highest prospective risk of blood-stage infections. Further studies are necessary to investigate the longevity of antibodies to PvRBPs.

The only longitudinal study to characterize the association between antibody responses to PvRBPs and clinical infection was conducted in a highly endemic area of PNG, showing that antibodies to PvRBP1a and PvRBP2b are strongly associated with reduced risk of clinical episodes among young children [16]. Our study with participants of all ages from a low *P*. *vivax* transmission setting enabled us to explore whether antibodies to PvRBPs were biomarkers of protective immunity in a broader age range. Antibody responses to PvRBP2b were consistently found to be strongly associated with protection in both cohorts. Although the significant protective associations with PvRBP2cNB were also found in both cohorts, PvRBP2cNB is a recombinant protein that does not include the functional binding domain. Therefore, the protective association with PvRBP2cNB is unlikely to be functional but could be due to the high correlation of antibody responses with PvRBP2b (rho = 0.76–0.77 P<0.001). Adjusting our statistical models for antibody levels to PvRBP2b completely accounted the for the protective association observed for PvRBP2cNB. Therefore, it is likely that the antibody response to PvRBP2cNB is a biomarker of immune status.

The TfR1-PvRBP2b invasion pathway is critical for *P*. *vivax* invasion into reticulocytes and monoclonal antibodies against PvRBP2b that block recombinant PvRBP2b binding to reticulocytes inhibit *P*. *vivax* invasion [21,27]. Given the very strong association of naturally acquired antibody responses to PvRBP2b with protection in Brazil, Thailand and PNG [16,41], it is likely that PvRBP2b antibodies are a functionally important aspect of naturally acquired anti-*P*. *vivax* immunity. As a consequence, PvRBP2b should be considered as a target for further development as a potential vaccine candidate.

There is a large body of evidence suggesting that protection against malaria is dependent on high levels of antibody concentrations [42]. Direct comparison of antibody levels to the same *P*. *falciparum* antigens between two cohorts of PNG 1–3 years old and 5–14 school-aged children indicated that a threshold level of antibodies might be required to be involved in protective immunity [42], in which IgG responses were substantially higher in the older cohort. The relatively low prevalence of *P*. *vivax* infections in our study (around 3% in both cohorts) may indicate a transmission level that may be too low to boost the residents immunity and indeed, compared to PNG adults, both Thai and Brazilian participants had low antibody titres. However, immune serum from participants with infection as determined by PCR in the study periods from Thailand and Brazil revealed a significant association of antibodies to PvRBP2b with clinical protection. The strength of the association with protection against clinical malaria was similar to that observed for PvRBP2b in the PNG children aged 1–3 years [16]. Collectively, these results show that relatively low levels of antibody to PvRBP2b may be sufficient for protection.

In summary, we showed that antibody responses to PvRBP1a, PvRBP2b, and PvRBP2cNB are suitable immunologic markers of exposure to *P*. *vivax* infection in malaria low-transmission settings and IgG responses to PvRBP2b are likely to protect the population against further clinical episodes. Further studies which include functional assays and antibody longevity measurements will be necessary to assess the potential of PvRBP2b as a vaccine candidate and to estimate the other PvRBPs as markers of exposure and/ or immunity.

## Supporting information

S1 Fig Receiver operator characteristics curves for antibody levels of PvRBPs to determine the risk of concurrent infection. (A) Antibody response to PvRBPs in Brazilian study. (B) Antibody response to PvRBPs in Thai study. Abbreviation: AUC = area under curve.
(TIF)

S1 Table Baseline characteristics of the study participants with infection. Note. For continuous quantities, Student t test was used to test for differences between the means of the groups, while chi-square test was performed to analyze the proportion differences of binary variables. P<0.05 was deemed significant. Abbreviation: molFOB = the molecular force of blood-stage infection.
(XLSX)

S2 Table Antibody levels of Brazilian and Thai cohort studies in comparison with antibody levels of PNG adults. Abbreviations: No = number, geom mean = Geometric mean, 95% CI = 95% confidence interval. *IgG levels multiplied by 1000. Values are in relative antibody units interpolated from standard curves using a 5PL logistic regression model.
(XLSX)

S1 Checklist STROBE checklist. (DOC)

## References

[1] Gething PW, Elyazar IRF, Moyes CL, Smith DL, Battle KE, et al (2012) A long neglected world malaria map: Plasmodium vivax endemicity in 2010. PLoS neglected tropical diseases 6: e1814 10.1371/journal.pntd.0001814 22970336 PMC3435256

[2] WHO (2017) World Malaria Report 2017: World Health Organization, Geneva.

[3] Rabinovich R, Drakeley C D A, Hall BF, Hay SI, Hemingway J, Kaslow DC, Noor A, Okumu F, Steketee R, Tanner M, Wells TNC, Whittaker MA, Winzeler EA, Wirth DF, Whitfield K, Alonso PL. (2017) malERA: An updated research agenda for diagnostics, drugs, vaccines, and vector control in malaria elimination and eradication. PLoS Med 14: e1002455 10.1371/journal.pmed.1002455 29190291 PMC5708606

[4] Guerra CA, Snow RW, Hay SI (2006) Defining the global spatial limits of malaria transmission in 2005. Adv Parasitol 62: 157–179. 10.1016/S0065-308X(05)62005-2 16647970 PMC3145102

[5] Bassat Q, Velarde M, Mueller I, Lin J, Leslie T, et al (2016) Key Knowledge Gaps for Plasmodium vivax Control and Elimination. Am J Trop Med Hyg 95: 62–71. 10.4269/ajtmh.16-0180 27430544 PMC5201224

[6] Garnham PC (1988) Swellengrebel lecture. Hypnozoites and ‘relapses’ in Plasmodium vivax and in vivax-like malaria. Trop Geogr Med 40: 187–195. 3055568

[7] Krotoski WA, Collins WE, Bray RS, Garnham PC, Cogswell FB, et al (1982) Demonstration of hypnozoites in sporozoite-transmitted Plasmodium vivax infection. Am J Trop Med Hyg 31: 1291–1293. 10.4269/ajtmh.1982.31.1291 6816080

[8] Robinson LJ, Wampfler R, Betuela I, Karl S, White MT, et al (2015) Strategies for understanding and reducing the Plasmodium vivax and Plasmodium ovale hypnozoite reservoir in Papua New Guinean children: a randomised placebo-controlled trial and mathematical model. PLoS medicine 12: e1001891 10.1371/journal.pmed.1001891 26505753 PMC4624431

[9] Mueller I, Galinski MR, Tsuboi T, Arevalo-Herrera M, Collins WE, et al (2013) Natural acquisition of immunity to Plasmodium vivax: epidemiological observations and potential targets. Advances in Parasitology 81: 77–131. 23384622 10.1016/B978-0-12-407826-0.00003-5

[10] Cowman AF, Tonkin CJ, Tham WH, Duraisingh MT (2017) The Molecular Basis of Erythrocyte Invasion by Malaria Parasites. Cell Host Microbe 22: 232–245. 10.1016/j.chom.2017.07.003 28799908 PMC12801281

[11] Malleret B, Li A, Zhang R, Tan KS, Suwanarusk R, et al (2015) Plasmodium vivax: restricted tropism and rapid remodeling of CD71-positive reticulocytes. Blood 125: 1314–1324. 10.1182/blood-2014-08-596015 25414440 PMC4401350

[12] Hester J, Chan ER, Menard D, Mercereau-Puijalon O, Barnwell J, et al (2013) De novo assembly of a field isolate genome reveals novel Plasmodium vivax erythrocyte invasion genes. PLoS neglected tropical diseases 7: e2569 10.1371/journal.pntd.0002569 24340114 PMC3854868

[13] Menard D, Chan ER, Benedet C, Ratsimbasoa A, Kim S, et al (2013) Whole Genome Sequencing of Field Isolates Reveals a Common Duplication of the Duffy Binding Protein Gene in Malagasy Plasmodium vivax Strains. PLOS Neglected Tropical Diseases 7: e2489 10.1371/journal.pntd.0002489 24278487 PMC3836732

[14] Carlton JM, Adams JH, Silva JC, Bidwell SL, Lorenzi H, et al (2008) Comparative genomics of the neglected human malaria parasite Plasmodium vivax. Nature 455: 757–763. 10.1038/nature07327 18843361 PMC2651158

[15] Galinski MR, Medina CC, Ingravallo P, Barnwell JW (1992) A reticulocyte-binding protein complex of Plasmodium vivax merozoites. Cell 69: 1213–1226. 10.1016/0092-8674(92)90642-p 1617731

[16] França CT, He W-Q, Gruszczyk J, Lim NTY, Lin E, et al (2016) Plasmodium vivax Reticulocyte Binding Proteins Are Key Targets of Naturally Acquired Immunity in Young Papua New Guinean Children. PLoS neglected tropical diseases 10: e0005014 10.1371/journal.pntd.0005014 27677183 PMC5038947

[17] Gupta ED, Anand G, Singh H, Chaddha K, Bharti PK, et al (2017) Naturally Acquired Human Antibodies Against Reticulocyte-Binding Domains of Plasmodium vivax Proteins, PvRBP2c and PvRBP1a, Exhibit Binding-Inhibitory Activity. J Infect Dis 215: 1558–1568. 10.1093/infdis/jix170 28379500 PMC5853946

[18] Han J-H, Lee S-K, Wang B, Muh F, Nyunt MH, et al (2016) Identification of a reticulocyte-specific binding domain of Plasmodium vivax reticulocyte-binding protein 1 that is homologous to the PfRh4 erythrocyte-binding domain. Scientific Reports 6: 26993 10.1038/srep26993 27244695 PMC4886630

[19] Ntumngia FB, Thomson-Luque R, Galusic S, Frato G, Frischmann S, et al (2018) Identification and immunological characterization of the ligand domain of Plasmodium vivax reticulocyte binding protein 1a. J Infect Dis. 218: 1110–1118. 10.1093/infdis/jiy273 29741629 PMC6107737

[20] Gruszczyk J, Lim NTY, Arnott A, He W-Q, Nguitragool W, et al (2016) Structurally conserved erythrocyte-binding domain in Plasmodium provides a versatile scaffold for alternate receptor engagement. Proceedings of the National Academy of Sciences of the United States of America 113: E191–200. 10.1073/pnas.1516512113 26715754 PMC4720341

[21] Gruszczyk J, Kanjee U, Chan LJ, Menant S, Malleret B, et al (2018) Transferrin receptor 1 is a reticulocyte-specific receptor for Plasmodium vivax. Science 359: 48–55. 10.1126/science.aan1078 29302006 PMC5788258

[22] Wright KE, Hjerrild KA, Bartlett J, Douglas AD, Jin J, et al (2014) Structure of malaria invasion protein RH5 with erythrocyte basigin and blocking antibodies. Nature 515: 427–430. 10.1038/nature13715 25132548 PMC4240730

[23] Drew DR, Beeson JG (2015) PfRH5 as a candidate vaccine for Plasmodium falciparum malaria. Trends Parasitol 31: 87–88. 10.1016/j.pt.2015.02.001 25704640

[24] Horuk R, Chitnis CE, Darbonne WC, Colby TJ, Rybicki A, et al (1993) A receptor for the malarial parasite Plasmodium vivax: the erythrocyte chemokine receptor. Science (New York, NY) 261: 1182–1184. 7689250 10.1126/science.7689250

[25] Miller LH, Mason SJ, Clyde DF, McGinniss MH (1976) The resistance factor to Plasmodium vivax in blacks. The Duffy-blood-group genotype, FyFy. The New England Journal of Medicine 295: 302–304. 10.1056/NEJM197608052950602 778616

[26] Ménard D, Barnadas C, Bouchier C, Henry-Halldin C, Gray LR, et al (2010) Plasmodium vivax clinical malaria is commonly observed in Duffy-negative Malagasy people. Proceedings of the National Academy of Sciences of the United States of America 107: 5967–5971. 10.1073/pnas.0912496107 20231434 PMC2851935

[27] Gruszczyk J, Huang RK, Chan LJ, Menant S, Hong C, et al (2018) Cryo-EM structure of an essential Plasmodium vivax invasion complex. Nature 559: 135–139. 10.1038/s41586-018-0249-1 29950717

[28] Rayner JC, Tran TM, Corredor V, Huber CS, Barnwell JW, et al (2005) Dramatic difference in diversity between Plasmodium falciparum and Plasmodium vivax reticulocyte binding-like genes. The American Journal of Tropical Medicine and Hygiene 72: 666–674. 15964948

[29] Pearson RD, Amato R, Auburn S, Miotto O, Almagro-Garcia J, et al (2016) Genomic analysis of local variation and recent evolution in Plasmodium vivax. Nat Genet 48: 959–964. 10.1038/ng.3599 27348299 PMC4966634

[30] Tetteh KK, Stewart LB, Ochola LI, Amambua-Ngwa A, Thomas AW, et al (2009) Prospective identification of malaria parasite genes under balancing selection. PLoS One 4: e5568 10.1371/journal.pone.0005568 19440377 PMC2679211

[31] Tran TM, Oliveira-Ferreira J, Moreno A, Santos F, Yazdani SS, et al (2005) Comparison of IgG reactivities to Plasmodium vivax merozoite invasion antigens in a Brazilian Amazon population. The American Journal of Tropical Medicine and Hygiene 73: 244–255. 16103583

[32] Ferreira AR, Singh B, Cabrera-Mora M, Souza ACMD, Marques MTQ, et al (2014) Evaluation of Naturally Acquired IgG Antibodies to a Chimeric and Non-Chimeric Recombinant Species of Plasmodium vivax Reticulocyte Binding Protein-1: Lack of Association with HLA-DRB1*/DQB1* in Malaria Exposed Individuals from the Brazilian Amazon. PLOS ONE 9: e105828 10.1371/journal.pone.0105828 25148251 PMC4141821

[33] Hietanen J, Chim-Ong A, Chiramanewong T, Gruszczyk J, Roobsoong W, et al (2016) Gene Models, Expression Repertoire, and Immune Response of Plasmodium vivax Reticulocyte Binding Proteins. Infection and Immunity 84: 677–685. 26712206 10.1128/IAI.01117-15 PMC4771344

[34] Nguitragool W, Karl S, White M, Koepfli C, Felger I, et al (2019) Highly heterogeneous residual malaria risk in western Thailand. Int J Parasitol 4: 30080–30083. 10.1016/j.ijpara.2019.01.004 30954453 PMC6996282

[35] Koepfli C, Colborn KL, Kiniboro B, Lin E, Speed TP, et al (2013) A High Force of Plasmodium vivax Blood-Stage Infection Drives the Rapid Acquisition of Immunity in Papua New Guinean Children. PLOS Negl Trop Dis 7: e2403 10.1371/journal.pntd.0002403 24040428 PMC3764149

[36] Kellar KL, Kalwar RR, Dubois KA, Crouse D, Chafin WD, et al (2001) Multiplexed fluorescent bead-based immunoassays for quantitation of human cytokines in serum and culture supernatants. Cytometry 45: 27–36. 11598944 10.1002/1097-0320(20010901)45:1<27::aid-cyto1141>3.0.co;2-i

[37] Lin E, Kiniboro B, Gray L, Dobbie S, Robinson L, et al (2010) Differential patterns of infection and disease with P. falciparum and P. vivax in young Papua New Guinean children. PloS One 5: e9047 10.1371/journal.pone.0009047 20140220 PMC2816213

[38] He WQ, Karl S, White M, Nuitragool W, Monteiro W, Kuehn A, Gruszczyk J, Franca C, Sattabongkot J, Lacerda M, Tham W, Mueller I Data from: Antibodies to Plasmodium vivax Reticulocyte Binding Protein 2b are Associated with Protection against P. vivax Malaria in Populations Living in Low Malaria Transmission Regions of Brazil and Thailand. 10.1371/journal.pntd.0007596 PMC6726234 31425514

[39] Ferreira MU, Castro MC (2016) Challenges for malaria elimination in Brazil. Malaria Journal 15: 284 27206924 10.1186/s12936-016-1335-1 PMC4875681

[40] Mueller I, Schoepflin S, Smith TA, Benton KL, Bretscher MT, et al (2012) Force of infection is key to understanding the epidemiology of Plasmodium falciparum malaria in Papua New Guinean children. Proceedings of the National Academy of Sciences of the United States of America 109: 10030–10035. 10.1073/pnas.1200841109 22665809 PMC3382533

[41] Franca CT, White MT, He WQ, Hostetler JB, Brewster J, et al (2017) Identification of highly-protective combinations of Plasmodium vivax recombinant proteins for vaccine development. eLife 6: e28673 28949293 10.7554/eLife.28673 PMC5655538

[42] Stanisic DI, Fowkes FJI, Koinari M, Javati S, Lin E, et al (2015) Acquisition of antibodies against Plasmodium falciparum merozoites and malaria immunity in young children and the influence of age, force of infection, and magnitude of response. Infection and Immunity 83: 646–660. 10.1128/IAI.02398-14 25422270 PMC4294228

