# Supplementary figures and images for: Antibodies to Plasmodium vivax reticulocyte binding protein 2b are associated with protection against P. vivax malaria in populations living in low malaria transmission regions of Brazil and Thailand
Source: PLoS Negl Trop Dis. 2019 Aug 19;13(8):e0007596. doi: 10.1371/journal.pntd.0007596 (PMC6726234; doi:10.1371/journal.pntd.0007596)

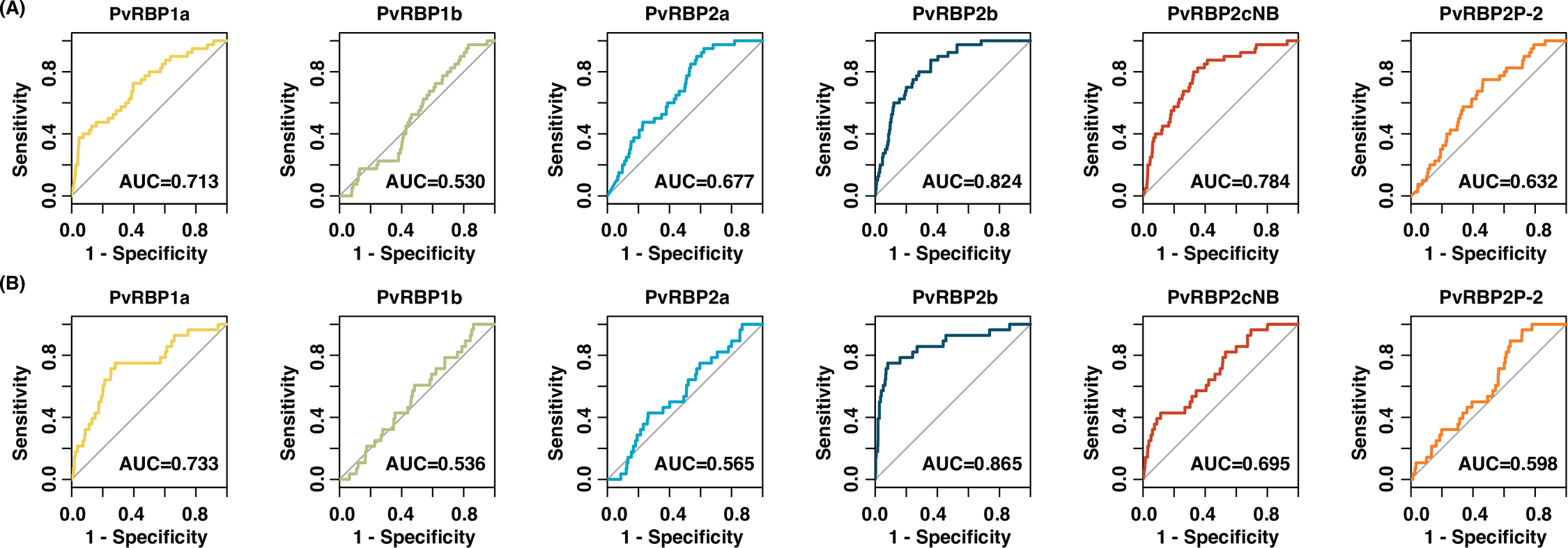

Supplement: S1 Fig — (A) Antibody response to PvRBPs in Brazilian study. (B) Antibody response to PvRBPs in Thai study. Abbreviation: AUC = area under curve. (TIF) [file pntd.0007596.s001.tif]
