# Supplementary material for: Antibodies to Plasmodium vivax reticulocyte binding protein 2b are associated with protection against P. vivax malaria in populations living in low malaria transmission regions of Brazil and Thailand
Source: PLoS Negl Trop Dis. 2019 Aug 19;13(8):e0007596. doi: 10.1371/journal.pntd.0007596 (PMC6726234; doi:10.1371/journal.pntd.0007596)
Supplement: S1 Checklist — (DOC) [file pntd.0007596.s004.doc]

STROBE Statement—Checklist of items that should be included in reports of ***cohort studies***

|  | Item No | Recommendation |
| --- | --- | --- |
| **Title and abstract** | 1 | (*a*) Use of samples from cohort study cited in abstract |
|  |  | (*b*) NA |
| Introduction | | |
| Background/rationale | 2 | Introduction paragraph 1-4 |
| Objectives | 3 | Introduction paragraph 5 |
| Methods | | |
| Study design | 4 | Section on cohort studies in Methods. Reference to Moteiro, Kuehn et al., in preparation for Brazilian study and Nguitragool et al., submitted for Thai study for details |
| Setting | 5 | Section on cohort study in Methods. Reference to Moteiro, Kuehn et al., in preparation for Brazilian study and Nguitragool et al., submitted for Thai study for details |
| Participants | 6 | (Section on cohort study in Methods. Reference to Moteiro, Kuehn et al., in preparation for Brazilian study and Nguitragool et al., submitted for Thai study for details |
|  |  | (*b*) NA |
| Variables | 7 | Sections on cohort study & statistical analyses in Methods. Reference to Moteiro, Kuehn et al., in preparation for Brazilian study and Nguitragool et al., submitted for Thai study for details |
| Data sources/ measurement | 8* | Sections on cohort study & statistical analyses & PvRBP conjugation and measurement of IgG responses in Methods. Reference to Moteiro, Kuehn et al., in preparation for Brazilian study and Nguitragool et al., submitted for Thai study for details |
| Bias | 9 | Sections on cohort study & statistical analyses in Methods. Reference to Moteiro, Kuehn et al., in preparation for Brazilian study and Nguitragool et al., submitted for Thai study for details |
| Study size | 10 | Defined by size of original study, Moteiro, Kuehn et al., in preparation for Brazilian study and Nguitragool et al., submitted for Thai study for details |
| Quantitative variables | 11 | Section on statistical analyses sections in Methods. |
| Statistical methods | 12 | Section on statistical analyses in Methods. Reference to Moteiro, Kuehn et al., in preparation for Brazilian study and Nguitragool et al., submitted for Thai study for details |
|  |  | (*b*) Section on statistical analyses in Methods |
|  |  | (*c*) Only participants with complete data were included in the analyses – see methods |
|  |  | (*d*) Only participants that completed follow-up were included in the analyses |
|  |  | (*e*) ND |
| Results | | |
| Participants | 13* | (a) 1163 participants from Brazilian study and 973 participants from Thai study in included – see section on study population |
|  |  | (b) see details in Reference to Moteiro, Kuehn et al., in preparation for Brazilian study and Nguitragool et al., submitted for Thai study for details |
|  |  | (c) incl. in Reference to Moteiro, Kuehn et al., in preparation for Brazilian study and Nguitragool et al., submitted for Thai study for details |
| Descriptive data | 14* | (a) Section on cohort study in Methods. Reference to Moteiro, Kuehn et al., in preparation for Brazilian study and Nguitragool et al., submitted for Thai study for details |
|  |  | (b) Only participants with complete data were included in the analyses – see methods |
|  |  | (c) see details in Reference to Moteiro, Kuehn et al., in preparation for Brazilian study and Nguitragool et al., submitted for Thai study for details |
| Outcome data | 15* | - Clinical episodes of P. vivax Malaria: Described in section of “Antibody responses to PvRBP2b predict reducted incidence of clinical malaria”. Additional details in Moteiro, Kuehn et al., in preparation for Brazilian study and Nguitragool et al., submitted for Thai study for details - Antibody titres: Described throughput the results sections |
| Main results | 16 | (*a*) A complete overview of un-adjusted and adjusted results is given in table 1-2 and supplementary tables S2-S4 |
|  |  | (*b*) Category boundaries for antibody measures included into Table S2 |
|  |  | (*c*) ND |
| Other analyses | 17 | Sub-analyses are described in details in the relevant results sections |
| Discussion | | |
| Key results | 18 | Paragraph 2,4,5,6,7 of discussion |
| Limitations | 19 | Paragraph 7 plus elsewhere in the discussion where appropriate |
| Interpretation | 20 | Throughout the discussion |
| Generalisability | 21 | Throughout the discussion |
| Other information | | |
| Funding | 22 | Specified in funding section |

*Give information separately for exposed and unexposed groups.

**Note:** An Explanation and Elaboration article discusses each checklist item and gives methodological background and published examples of transparent reporting. The STROBE checklist is best used in conjunction with this article (freely available on the Web sites of PLoS Medicine at http://www.plosmedicine.org/, Annals of Internal Medicine at http://www.annals.org/, and Epidemiology at http://www.epidem.com/). Information on the STROBE Initiative is available at http://www.strobe-statement.org.
